# Dog saliva – an important source of dog allergens

**DOI:** 10.1111/all.12130

**Published:** 2013-03-07

**Authors:** N Polovic, K Wadén, J Binnmyr, C Hamsten, R Grönneberg, C Palmberg, N Milcic-Matic, T Bergman, H Grönlund, M van Hage, Reto Crameri

**Affiliations:** 1 Department of Medicine Solna, Clinical Immunology and Allergy Unit, Karolinska InstitutetStockholm, Sweden; 2 Department of Biochemistry, Faculty of Chemistry, University of BelgradeBelgrade, Serbia; 3 Center for Inflammatory Diseases, Karolinska InstitutetStockholm, Sweden; 4 Department of Medicine Solna, Respiratory Medicine Unit, Karolinska InstitutetStockholm, Sweden; 5 Department of Medical Biochemistry and Biophysics, Karolinska InstitutetStockholm, Sweden; 6 Department of Dermatology, Faculty of Veterinary Medicine, University of BelgradeBelgrade, Serbia; 7 Department of Clinical Neuroscience, Therapeutic Immune Design Unit, Karolinska InstitutetStockholm, Sweden

**Keywords:** allergen, *Canis familiaris*, diagnosis, dog allergy, saliva

## Abstract

**Background:**

Allergy to dog (*Canis familiaris*) is a worldwide common cause of asthma and allergic rhinitis. However, dander extract in routine diagnostics is not an optimal predictor of IgE-mediated dog allergy. Our objective was to evaluate saliva as an allergen source for improved diagnostics of allergy to dog.

**Methods:**

IgE-binding proteins in dog saliva and dander extract were analysed by immunoblot and mass spectrometry (LC-MS/MS) using pooled or individual sera from dog-allergic patients (*n* = 13). Sera from 59 patients IgE positive to dander and 55 patients IgE negative to dander but with symptoms to dog were analysed for IgE against saliva and dander by ELISA. Basophil stimulation with dog saliva and dander extract was measured by flow cytometry among three dog-allergic patients. Additionally, IgE-binding protein profiles of saliva from different breeds were investigated by immunoblot.

**Results:**

Greater number and diversity of IgE-binding proteins was found in saliva compared to dander extract and varied among dog breeds. In saliva, Can f 1, 2, 3 and 6 were identified but also four new saliva allergen candidates. The majority of the 59 dog dander–positive sera (*n* = 44) were IgE positive to dog saliva. Among patients IgE negative to dander, but with symptoms to dog, 20% were IgE positive to saliva. The biological activity of saliva was confirmed by basophil degranulation.

**Conclusions:**

Dog saliva is an allergen source for improved diagnostics of dog allergy. The IgE-binding protein profile of saliva from different dogs varies.

Allergy to dog (*Canis familiaris*) is a worldwide problem that affects 5–10% of the adult population 1–3 and serves as a triggering factor in children and adults who suffer from asthma and allergic rhinitis 1, 2. It has been estimated that sensitisation to dog, confirmed by skin prick test, occurs in children with a physician-diagnosed asthma, rhinitis or eczema in up to 34%, 33% and 21%, respectively, in Sweden 4.

Commercial dander extracts are routinely used for diagnosis of allergy to dog, both *in vitro* and *in vivo*. Dander is the preferable source for dog allergen extract preparations 5, 6. However, the outcome of *in vitro* IgE determinations and skin tests largely depends on the quality of the extracts 6. Clinical experience reveals that tests with commercially available dog allergen extracts occasionally show only slightly positive or even negative results, although the tested patients clearly exhibit dog-related symptoms 7, 8. Furthermore, dog dander extracts might be contaminated with mite allergens that potentially cause false positive skin prick test results 6, 9.

Animal saliva is known as a common source of allergens. The major cat allergen, Fel d 1, is a tear, skin and salivary protein. Fel d 1 is produced by lacrimal and sublingual glands and subsequently transferred to fur by licking 10. Rat 11 and rabbit 12 saliva are reported as sources of numerous allergens that differ in SDS-PAGE profiles from analogous dander extract allergens. Five major allergens in rat saliva of molecular weights of 21.5 kDa or less have been identified by immunoblot 11. In rabbit saliva, 12 IgE-binding proteins have been identified. Two of them have by N-terminal sequencing shown to belong to the lipocalin family, while one was identified as the Fel d 1 homologue uteroglobin 12.

Can f 1, Can f 2 together with serum albumin (Can f 3) and Can f 4 are known allergens from dog, although other possible candidates have been observed in immunoblot 13, 14. The two allergens Can f 1 and Can f 2 that belong to the lipocalin protein family are recognised as important allergens and are both found in dander and saliva 15, 16. Can f 1 binds more than 50% and Can f 2 one-third of IgE from dog-allergic patients 17. Can f 3 and Can f 4 are less important allergens that cross-react with allergens from other furry animals. In 2009, prostatic kallikrein (Can f 5) derived from dog urine was identified as a major allergen 14. A homologous protein was also detected in dog dander. Recently, the lipocalin Can f 6 was reported as an allergen cross-reactive with cat and horse 18. Despite being a major allergen, Can f 1 alone is not sufficient for diagnosis of dog allergy 16, 17.

The aim of this study was to evaluate dog saliva as a potential source of allergens for improved diagnosis of allergy to dog.

## Methods

### Subjects

All subjects were recruited from the Karolinska University Hospital, Stockholm, Sweden (Table 1).

**Table 1:** Subjects enrolled in the study

Subjects	Number of individuals and IgE antibody levels to dog dander in ImmunoCAP (e5); (range kU_A_/l)	Age (years): median; (range)	Gender: M/F	Included in
Dog-allergic patients	13 (4.9–99)	39 (20–59)	7/5	Immunoblot
Dog-allergic patients	3 (0.1–2.6)	27 (25–27)	1/2	Basophil activation
Dog dander-sensitised patients	59 (1.22–100)	20 (1–69)	31/28	ELISA
Patients with suspected dog allergy	55 (<0.1)	32 (10–81)	22/33	ELISA
Non-dog dander-sensitised individuals (controls)	67 (<0.1)	36 (32–57)	58/9	ELISA

Sera from 13 dog-allergic patients (median IgE level, 18 kU_A_/l to dog; range, 4.9–99 kU_A_/l; e5, ImmunoCAP System; Phadia AB, Uppsala, Sweden) were used individually or as a pool (2.5 kU_A_/l) to test IgE binding to proteins from dog saliva and dog dander by immunoblotting. The patients were selected on the basis of IgE to dog dander and a positive case history of dog allergy.

Sera from 59 patients sensitised to dog dander (median IgE level, 26 kU_A_/l; range, 1.22–100 kU_A_/l) were selected only on IgE to dog dander. The twenty-first patient sera were used to set up and validate the dog dander ELISA against ImmunoCAP. Then, the 59 patients and 55 patients IgE negative to dog dander but with symptoms to dog were tested in ELISA for IgE against dog saliva and dog dander proteins. In addition, sera from 67 non-dog dander-sensitised individuals, recruited on the basis of lacking IgE (<0.1 kU_A_/l) to dog dander extract with negative skin prick test to dog dander extract and having no symptoms to dog, and IgE myeloma (1000 kU/l) were used as controls.

Sera from three patients with a doctor's diagnosis of dog allergy (e5; 0.1, 2.2 and 2.6 kU_A_/l, respectively) were used in a basophil activation assay. The study was approved by the local ethics committee.

### Dog dander and saliva

Dog dander (skin and hair extract) was obtained from Allergon AB (Ängelholm, Sweden). Dog saliva was collected from 14 dogs of 11 different breeds (Fig. 5). The dogs were clinically healthy attending the clinic for small animals at the Faculty of Veterinary Medicine, University of Belgrade for their annual vaccination. A dog saliva pool (of the 14 samples) and the dog dander extract were prepared as described 16.

The study was approved by the local ethics committee for animal welfare.

### SDS-PAGE and immunoblotting

Proteins in individual dog saliva samples, dog saliva pool and dog dander extracts were separated by SDS-PAGE and stained using Coomassie brilliant blue (CBB) or analysed by immunoblot under reducing conditions. Twelve microgram protein per lane was analysed using serum from 13 dog-allergic patients, each diluted to 2.5 kU_A_/l. For details, see Data S1

### 2D PAGE

Proteins in the dog saliva pool and dog dander extract (100 μg) were separated by 2D PAGE and detected using a serum pool from 13 dog-allergic patients (2.5 kU_A_/l). For details, see Data S1.

### Protein identification by mass spectrometry

Protein spots in 2D gels were subjected to tryptic in-gel digestion followed by LC-MS/MS analysis and database searches. For details, see Data S1.

### ELISA

Sera were diluted in dilution buffer to an IgE antibody concentration in the range of 0.5–5 kU_A_/l, suitable for the indirect ELISA measurement. All analyses were run in duplicates. IgE values to dog dander and dog saliva were considered positive when the IgE responses exceeded mean + 3SD of the controls (OD ≥ 0.085 for dog dander, and OD ≥ 0.123 for dog saliva). The IgE myeloma controls were negative, saliva (OD 0.029) and dander (OD 0.015). For details, see Data S1.

### Basophil activation test

Allergen-specific basophil degranulation was analysed by monitoring the basophil activation markers CD203c and CD63 19. For details, see Data S1.

### Statistical analysis

Statistical analysis was performed using the software Origin 7.0 (OriginLab, Northampton, MA, USA). Spearman rank was used for correlation analysis. *P* < .05 was considered significant.

## Results

### Dog-allergic patients recognise salivary proteins in IgE immunoblot

**Figure 1:**
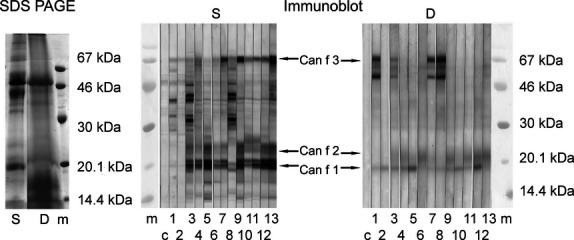
SDS–PAGE and immunoblot analyses of dog saliva (S) pool (*n* = 14) and dog dander (D) extract (allergon). Immunoblot was developed with single dog-allergic patient's sera. m, Molecular weight markers; c, control (buffer); lanes 1–13, patient sera.

There was a greater abundance and diversity of IgE-binding proteins in dog saliva compared to dog dander extract (Fig. 1). In dog dander extract, most of the patients recognised rather few protein bands at positions corresponding to sizes of already described dog allergens. In contrast, the dog saliva pool revealed at least 12 IgE-binding proteins and several of those were of molecular weight sizes not recognised in the dog dander extract (Fig. 1).

### Identification of IgE-binding proteins in saliva and dander extracts

**Figure 2:**
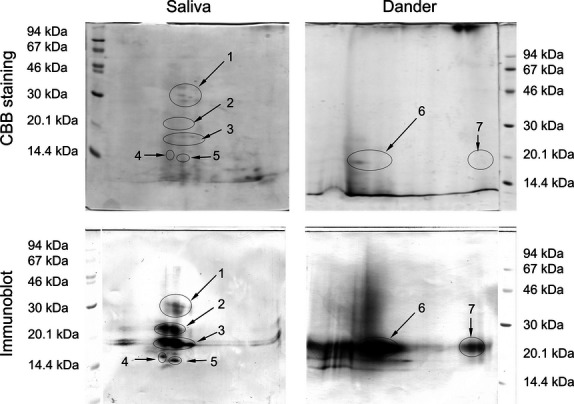
2D PAGE of dog saliva pool (*n* = 14) and dog dander extract (allergon). The pI range was 3–10, left to right. Immunoblot was developed with a serum pool from 13 dog-allergic patients. Protein spot regions analysed by mass spectrometry are labelled 1–7. For protein identifications (Table 2).

The IgE-binding protein profile in 2D PAGE using the dog-allergic patient serum pool revealed some resemblance between dog dander extract and saliva. The IgE-binding proteins appear to have mainly acidic pI values (pI about 5). However, salivary proteins showed a greater microheterogeneity regarding isoelectric point pattern (Fig. 2).

Mass spectrometry was applied to identify the most prominent IgE-binding proteins in both dog dander extract and saliva. Coomassie brilliant blue staining of 2D gels revealed seven spot regions corresponding to distinct IgE-binding areas in the immunoblot, five in the saliva gel and two in the dander gel (Fig. 2). These spots were subjected to trypsin in-gel digestion followed by protein identification using liquid chromatography tandem mass spectrometry (LC-MS/MS) sequencing of peptides and database searches.

From the saliva gel, Can f 3 was identified in all five spots, Can f 1 in three spots, while Can f 2 and Can f 6 were identified in spot 2 only. Additionally, BPI fold-containing family A member 2 isoform 1 (BPIFA2) was identified in all five spots, mucin-5B and angiopoietin-related protein 5-like (ANGPTL5) in four spots, the IgA heavy chain constant region in three spots and BPI fold-containing family A member 1 (BPIFA1) in spot 3 only. Can f 1–4, Can f 6 and BPIFA1 were identified in the two spots from the dander extract gel. See Table 2 for further information on protein hits, their accession numbers and scores.

**Table 2:** *Canis lupus familiaris* proteins identified by LC-MS/MS and Mascot database searches after in-gel tryptic digestion of proteins in dog saliva and dog dander extract resolved by 2D PAGE

Spot	Protein	Accession*	Protein score†	Nominal Mass	No. of peptide matches	No. of distinct sequences
1	Angiopoietin-related protein 5-like	gi|73968855	307	31683	9	5
	BPI fold-containing family A member 2 isoform 1‡	gi|73991578	277	27194	14	9
	Mucin-5B	gi|345783652	163	545794	6	6
	IgA heavy chain constant region	gi|598107	155	38168	6	5
	Albumin§	gi|3319897	140	67857	7	7
2	BPI fold-containing family A member 2 isoform 1‡	gi|73991578	347	27194	22	8
	Mucin-5B	gi|345783652	197	545794	16	15
	Major allergen Can f 1 precursor	gi|50978938	162	19407	8	6
	Serum albumin precursor§	gi|55742764	130	70556	8	8
	Angiopoietin-related protein 5-like	gi|73968855	107	31683	3	2
	Allergen Fel d 4-like¶	gi|73971966	97	22096	5	5
	Minor allergen Can f 2 precursor	gi|50978944	63	20445	1	1
3	Serum albumin precursor§	gi|55742764	167	70556	12	10
	BPI fold-containing family A member 1	gi|73992235	157	26872	5	3
	BPI fold-containing family A member 2 isoform 1‡	gi|73991578	132	27194	9	5
	IgA heavy chain constant region	gi|598107	111	38168	5	5
	Major allergen Can f 1 precursor	gi|50978938	86	19407	8	6
	Angiopoietin-related protein 5-like	gi|73968855	42	31683	2	2
4	Serum albumin precursor§	gi|55742764	249	70556	15	15
	Mucin-5B	gi|345783652	120	545794	12	10
	BPI fold-containing family A member 2 isoform 1‡	gi|73991578	61	27194	6	3
	Angiopoietin-related protein 5-like	gi|73968855	56	31683	1	1
5	Albumin§	gi|3319897	217	67857	11	11
	IgA heavy chain constant region	gi|598107	95	38168	9	5
	Mucin-5B	gi|345783652	68	545794	9	9
	BPI fold-containing family A member 2 isoform 1	gi|73991578	57	27194	2	2
	Major allergen Can f 1 precursor	gi|50978938	34	19407	2	2
6	Allergen Can f 4 precursor	gi|300116720	185	19450	11	9
	Serum albumin precursor§	gi|55742764	169	70556	9	9
	BPI fold-containing family A member 1	gi|73992235	160	26872	3	3
	Major allergen Can f 1 precursor	gi|50978938	89	19407	2	2
	Allergen Fel d 4-like¶	gi|73971966	78	22096	3	3
	Minor allergen Can f 2 precursor	gi|50978944	40	20445	1	1
7	Major allergen Can f 1 precursor	gi|50978938	107	19407	5	4
	Allergen Can f 4 precursor	gi|300116720	61	19450	6	6
	Allergen Fel d 4-like¶	gi|73971966	45	22096	2	2
	Albumin – dog (fragment)§	gi|2147092	38	30901	2	2

* Database NCBInr November 2012.

† Mascot Search (http://www.matrixscience.com).

‡ BPI-like protein family includes forms of parotid secretory protein.

§ Allergenic protein Can f 3.

¶ Allergenic protein Can f 6.

### IgE reactivity to dog saliva

**Figure 3:**
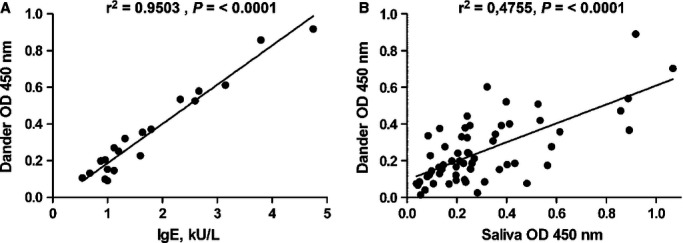
IgE reactivity. (A) Correlation between IgE reactivity to dog dander by ELISA (*y-*axis) and ImmunoCAP (e5) (*x-*axis) (*n* = 20); (B) correlation between IgE reactivity in ELISA to dog dander *(y-*axis) and dog saliva (*x-*axis) (*n* = 59); OD – optical density, *r*^2^ – correlation factor.

The IgE reactivity to dog dander in ELISA was compared with ImmunoCAP (e5) using sera from 20 dog dander-sensitised individuals. All sera were IgE positive to dog dander in ELISA (OD; median, 0.262; range, 0.090–0.918). A good linear correlation was obtained for the IgE reactivity in ELISA and ImmunoCAP (*r*^2^ = 0.95, *P* < 0.0001) (Fig. 3A).

In the next step, the 59 dog dander-sensitised patients were analysed for IgE to dog dander and saliva by ELISA. The majority, 44 patients, was IgE positive to dog saliva (OD; median, 0.276; range, 0.123–0.891). Approximately half of the sera recognised both dander and salivary proteins to an equal extent. Some subjects (23/53; 39%) had a higher IgE reactivity to saliva than to dander. The correlation between IgE responses to saliva and dander in ELISA was *r*^2^ = 0.48 (*P* < 0.0001) (Fig. 3B). Interestingly, 11 of 55 patients (20%), who were IgE negative to dog dander in ImmunoCAP and ELISA but had symptoms to dog, were IgE positive to saliva (OD; median, 0.139; range, 0.125–0.188).

### Basophil activation

**Figure 4:**
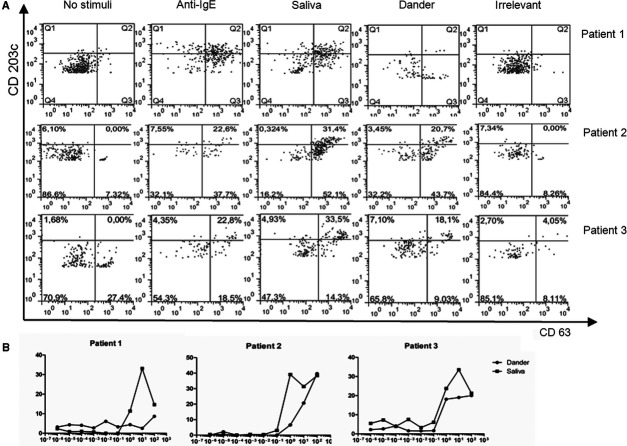
(A) Basophil degranulation: double-stained (CD 63, CD 203c) dot plot: from left to right unstimulated cells, positive control, stimulated with 10 μg/ml of dog saliva, dog dander extract and irrelevant allergen (rLep d 7), respectively. (B) Per cent upregulated basophils (*y-*axis) induced by serial dilutions of dog dander extract and saliva (*x-*axis); in blood from three representative dog-allergic patients.

A degranulation upon stimulation with dog saliva could be seen in all three patients. One patient was low- or nonresponding to dog dander (Fig. 4). No activation was seen upon stimulation of the two controls (a nonallergic individual and a cat-allergic patient without IgE to dog) (data not shown).

### IgE-binding profile of dog saliva

**Figure 5:**
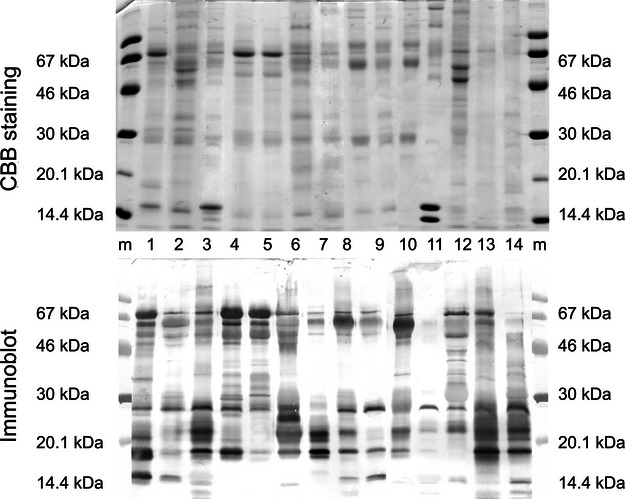
SDS-PAGE and immunoblot analysis of saliva from different dog breeds developed with pool of 13 dog-allergic patients' sera. m, Molecular weight markers; 1. German Wirehaired Pointer, male; 2. German Shorthaired Pointer, male; 3. German Shepherd Dog, male; 4. Cocker Spaniel, male; 5. Cocker Spaniel, bitch; 6. Doberman Pinscher, male; 7. Doberman Pinscher, bitch; 8. Neapolitan Mastiff, male; 9. Dogue de Bordeaux, male; 10. Saint Bernard's Dog, bitch; 11. Golden Retriever, male; 12. Pekingese, bitch; 13. Mixed breed, bitch; 14. Mixed breed, bitch.

In most of the samples, a considerable number (>12) of IgE-binding proteins with molecular weights ranging from 14 to 67 kDa could be detected. The concentration differed among the various allergens in the samples and some dogs, including the Golden Retriever and Dogue de Bordeaux, displayed fewer IgE-binding components compared to dogs of other breeds (Fig. 5). In 12 of 14 saliva samples, a band with a similar size as for Can f 1, that is, about 18 kDa, was present but the abundance was not evenly distributed. The band was not detected in sample 12 of Pekingese dog. The band at about 30 kDa, corresponding to the size of BPIFA2, was present in all saliva samples (Fig. 5).

## Discussion

In this study, we have investigated dog saliva as a source of dog allergens. Our results reveal that there are at least 12 protein bands in dog saliva that are recognised by IgE of dog-allergic patients. Furthermore, based on biochemical behaviour, electrophoresis and immunoblots, we conclude that dog saliva has a greater potential than dander as an allergen source.

Traditionally, dander, fur and skin are considered the best sources for animal allergen extract preparation 5. Such knowledge is based on reports in which animal dander, saliva and urine have been compared regarding IgE binding *in vitro* 20. However, the number and diversity of IgE-binding proteins in animal dander seems to be limited, especially in the case of dog allergens. There is a strong demand for new allergen sources and identification of new dog allergens. The current trend in improving allergy diagnostics involves component-resolved diagnostics of panels of allergens 21. In line with this goal, Can f 5 and Can f 6 have recently been described as dog allergens 14, 18.

Regarding the IgE-binding protein profile in dog dander extract, most of the patients recognised protein bands that corresponded to sizes of described dog allergens, whereas in the saliva pool, at least 12 IgE-binding proteins could be detected with molecular weights ranging from 14 to 67 kDa. We show the presence of Can f 1, Can f 2, Can f 3 and Can f 6 in dog saliva by LC-MS/MS analysis. The presence of Can f 1 and Can f 2 in saliva is known 10 and the level is higher than in urine or faeces 22. We identified four novel IgE-binding proteins from saliva: BPIFA2, Mucin-5B, ANGPTL5 and the IgA heavy chain constant region. BPIFA2, formerly known as Paratoid secretory protein, belongs to the PLUNC family, which is involved in mucosal host defence (BPI/LBP/PLUNC superfamily) 23. Mucin-5B is a mucus glycoprotein important for lubricating epithelial surfaces 24. ANGPTL5 belongs to the ANGPTL family involved in angiogenesis and triglyceride metabolism 25. Dog IgA is an interesting hit in analogue to cat IgA (Fel d 5) an allergen previously identified by us 26. However, these four proteins may be co-migratory proteins unrelated to the specific IgE binding at the spot regions analysed and further analysis is needed.

The values obtained in our dog dander–coated ELISA correlated significantly with IgE to dog dander measured in ImmunoCAP, indicating similar allergenic content in these extracts. However, there was only a modest correlation between IgE reactivity to dog saliva and dog dander, where several patients had higher IgE values to saliva than to dander. This work thus demonstrates that there is a difference in allergen content of these two dog allergen sources, suggesting saliva as an important addition to dander proteins in allergy diagnostics.

We also observed that one-fifth of patients with symptoms to dog, but lacking IgE antibodies to dander, were IgE positive to saliva. Dog dander may contain saliva in low amounts. However, these amounts seem nonsufficient to elicit an IgE response to saliva. The results have important clinical implications because diagnosis of dog allergy in daily practice relies on the clinical history of the patient together with diagnostics based on dog dander extract. Thus, the shortcomings of dog dander extracts can be improved by adding dog saliva.

Basophil degranulation upon stimulation with dog saliva was seen in all patients, emphasising that saliva is an allergen source. Dog saliva gave rise to higher or similar basophil activation than dog dander. Interestingly, one of these patients reacted poorly to dog dander extract (Fig. 4).

When investigating saliva from different dog breeds, we noted that there is a great variation in the IgE-binding profile. Interestingly, we found fewer IgE-binding proteins in saliva from some dogs, including the Golden Retriever and Dogue de Bordeaux, than in saliva from other dogs. Even though the number of samples is limited, the results indicate that some dogs could be better tolerated by allergic subjects than others. In several studies, it has been shown that the presence and quantity of Can f 1 can differ among dander extracts from different dogs 8, 27. One study showed that Can f 1 is absent or less abundant in some common breeds including Golden Retriever 8, while another study showed that Can f 1 levels in Labrador Retriever dander extracts were significantly lower than in extracts from other breeds 27. It is well established that gender, age and eczema status influence the concentration of Can f 1 quantities in hair, but it is not clear if individual differences are more relevant than breed-specific factors 8, 27.

In conclusion, this study reports on dog saliva as an important source of dog allergens. About one-fifth of patients with symptoms to dog but lacking IgE antibodies to dog dander were IgE positive to saliva. A greater abundance and diversity of IgE-binding proteins was found in dog saliva compared to dog dander extract, as well as differences in saliva allergen profiles from different dogs. Dog saliva is therefore a promising allergen source for improved diagnosis of dog allergy.

## Abbreviations

CBB: Coomassie brilliant blue
LC: liquid chromatography
MS/MS: tandem mass spectrometry
