# Supplementary material for: Dog saliva – an important source of dog allergens
Source: Allergy. 2013 Mar 7;68(5):585–92. doi: 10.1111/all.12130 (PMC3652036; doi:10.1111/all.12130)
Supplement: Supplementary file 1 [file all0068-0585-SD1.pdf]

Dog saliva – an important source of dog allergens

N. Polovic[1,2]*, K. Wadén[1]*, J. Binnmyr[1]*, C. Hamsten[1,3], R. Grönneberg[4], C. Palmberg[5], N. Milcic-Matic[6], T. Bergman[5], H. Grönlund[1,7] & M. van Hage[1]

[1]Department of Medicine Solna, Clinical Immunology and Allergy Unit, Karolinska Institutet, Stockholm, Sweden, [2]Department of Biochemistry, Faculty of Chemistry, University of Belgrade, Belgrade, Serbia, [3]Center for Inflammatory Diseases, Karolinska Institutet, Stockholm, Sweden, [4]Department of Medicine Solna, Respiratory Medicine Unit, Karolinska Institutet, Stockholm, Sweden, [5]Department of Medical Biochemistry and Biophysics, Karolinska Institutet, Stockholm, Sweden, [6]Department of Dermatology, Faculty of Veterinary Medicine, University of Belgrade, Belgrade, Serbia, [7]Department of Clinical Neuroscience, Therapeutic Immune Design Unit, Karolinska Institutet, Stockholm, Sweden

* These authors contributed equally to this work.

Correspondence: Marianne van Hage, MD, PhD, Department of Medicine Solna, Clinical Immunology and Allergy Unit, Karolinska Institutet , Stockholm, Sweden, Phone: +46-8-517 75942, Fax: +46-8- 33 57 24, E-mail: marianne.van.hage@ki.se

Supporting information

Methods

SDS-PAGE and immunoblotting

Protein concentrations were determined by the BCA™ protein assay (Pierce, Rockford, IL, USA). SDS-PAGE of individual dog saliva samples, dog saliva pool and dog dander extracts were analysed by immunoblot under reducing conditions. Twelve µg of proteins per lane were

resolved by 12% PAGE and electroblotted to Immobilon^{TM} polyvinylidene difluoride (PVDF) transfer membranes (Millipore, Billerica, MA, USA) following manufacturer's instruction.

A total of 250 µL serum from 13 dog allergic patients, each diluted to 2.5 kU_A/l in phosphate-buffered saline (PBS) containing 0.05% Tween 20 and 0.2% BSA, pH 7.4 (dilution buffer), was added to PVDF strips, blocked 1 h in Blocking Buffer (PBS 0.5% Tween 20 and 0.2% BSA, pH 7.4) and incubated overnight. Thereafter, rabbit anti-human IgE (1:2000; kind gift from Phadia AB) and subsequently alkaline phosphatase conjugated goat anti-rabbit IgG antibodies (1:1000; Jackson ImmunoResearch Laboratories, West Grove, PA, USA) were added and incubated for 2 h at room temperature, followed by addition of nitro blue tetrazolium (NBT) and 5-bromo-4-chloro-3-indolyl phosphate (BCIP) substrates (BioRad Laboratories, Hercules, CA, USA).

2D PAGE

Desalted dog saliva pool or dog dander proteins (100 µg) were loaded on 7.7 cm ZOOM strips (pH 3–10) (Invitrogen, Carlsbad, CA, USA) for the first dimension separation, followed by separation in the second dimension using homogeneous 12% SDS-PAGE and Coomassie Brilliant Blue (CBB) staining or electroblotting. After electroblotting, the membranes were blocked as above and incubated overnight with 9 ml of the pool containing serum from 13 dog allergic patients (2.5 kU_A/l). Bound IgE was detected as described above.

Protein identification by mass spectrometry

For protein identification by tandem mass spectrometry (MS/MS) sequencing of tryptic peptides, protein spots were cut from the 2D gel after staining with CBB and subjected to trypsin in-gel digestion. The CBB-stained protein spots were digested using a MassPREP robotic protein handling system (Waters, Milford, MA, USA). Briefly, washing was carried out in 50 mM ammonium bicarbonate containing 50% acetonitrile. The protein was reduced (dithiothreitol) and alkylated (iodoacetamide) followed by in-gel digestion with 0.3 µg Modified Trypsin (Promega, Madison, WI, USA) in 50 mM ammonium bicarbonate for 5 h at 40°C. The tryptic peptides were extracted with 1% formic acid/ 2% acetonitrile, followed by extraction with 50% acetonitrile twice. The acetonitrile was evaporated and the peptide extract concentrated to 5-10 µl under a stream of nitrogen.

MS/MS data was acquired with a QTOF Premier API instrument (Waters) equipped with the standard Z-spray source. Sample was introduced via a nanoAcquity liquid chromatography system (Waters) and electrosprayed using a PicoTip emitter (SilicaTip, New Objective, Woburn, MA, USA). Samples were desalted for 1 min using a Waters Symmetry C_{18} column (5 µm particles; 180 µm x 2 cm) using 0.1% formic acid at 15 µl/min, followed by separation using a Waters BEH C_{18} column (1.7 µm particles; 75 µm x 15 cm) employing a solvent system of 0.1% formic acid (solvent 1) and acetonitrile / 0.1% formic acid (solvent 2). The peptides were eluted with a linear gradient: 3-60% solvent 2 for 30 min at 300 nl/min. The capillary voltage was 2.3 kV, while the voltages of the sampling cone and the extraction cone were 40 and 2.5 V, respectively. The collisions gas was argon. DDA (Data Dependent Acquisition) was employed over a mass range 300-2000 *m/z* with a scan time of 1 min. Peptides of interest were detected through automated switching between MS and MS/MS. The

collision energy in MS/MS was automatically varied between 25 and 45 eV, depending on the mass and charge states of the peptides. Data analysis was performed using ProteinLynx Global SERVER 2.3 (PLGS 2.3, Waters) software and MassLynx peptide sequence software 4.0 (Waters). The lockmass-reference of Glu fibrinopeptide B (Sigma) was used for mass scale correction when processing the data in PLGS 2.3 software. Finally, the MS/MS data was submitted to Mascot (www.matrixscience.com) searches in the NCBInr (20121111) database. Search parameters were: up to one missed cleavage allowed, modifications applied were carbamidomethyl (Cys) and oxidation (Met), monoisotopic mass mode, peptide tolerance plus/minus 1.2 Da, MS/MS tolerance plus/minus 0.6 Da, and peptide charge states 2+ and 3+.

ELISA

Microtiter plates (96 wells, Nunc, Roskilde, Denmark) were coated overnight with 1 µg of dog dander or dog saliva proteins per well, followed by blocking (PBS containing 1% BSA and 0.5% Tween 20, pH 7.4) for 2 h at room temperature. Sera were diluted in dilution buffer to an IgE antibody concentration in the range of 0.5-5 kU_A/l suitable for the indirect ELISA measurement. After incubation for 1 h, rabbit anti-human IgE (1:2000; Phadia AB) and then horseradish peroxidase-conjugated donkey anti-rabbit antibodies (1:1000; Jackson ImmunoResearch Laboratories, West Grove, PA, USA) were added. Finally, o-Phenylenediamine dihydrochloride substrate (Sigma-Aldrich, St. Louis, MO, USA) was added and incubated for 1 h. The absorbance, optical density (OD), was measured at 450 nm.

Basophil activation test

Allergen-specific basophil degranulation was analysed by monitoring the basophil activation markers CD203c and CD63 (19). Briefly, 10-fold serial dilutions of dog saliva or dog dander

(10 µg/mL to 10^{-7}µg/ml), irrelevant allergen (rLep d 7), medium (negative control) and 2 µl of 0.5mg/ml rabbit anti-human IgE (kind gift from Phadia AB) and 30 µL of B-CCR STCON (Skafte medlab, Onsala, Sweden) (positive controls) were added to venous blood samples from three patients with a diagnosis of dog allergy. The samples were further incubated with anti-CD63 and anti-CD203c monoclonal antibodies (Immunotech, Marseille, France) and analyzed by flow cytometry using a FACS Calibur flow cytometer (BD Biosciences, San Jose, CA, USA) and data was analyzed using FlowJo (Treestar, Ashland, OR, USA). Basophils were identified by gating for CD203c positive cells and the magnitude of allergen-activation was calculated and expressed as the percentage of upregulated CD63 and 203c among the gated basophils compared with negative control sample. Two controls were included, serum from one non-allergic individual and from one cat allergic patient without IgE to dog (data not shown).
